# Development and external validation of a nomogram for predicting postoperative pneumonia in aneurysmal subarachnoid hemorrhage

**DOI:** 10.3389/fneur.2023.1251570

**Published:** 2023-09-04

**Authors:** Xiao Jin, Shijia Wang, Chengwei Zhang, Song Yang, Lejing Lou, Shuyao Xu, Chang Cai

**Affiliations:** ^1^Department of Respiratory and Critical Care Medicine, The First Affiliated Hospital of Wenzhou Medical University, Wenzhou, China; ^2^Department of Neurosurgery, The First Affiliated Hospital of Wenzhou Medical University, Wenzhou, China

**Keywords:** postoperative pneumonia, aneurysmal subarachnoid hemorrhage, Medical Information Mart for Intensive Care, nomogram, external validation

## Abstract

**Background:**

Postoperative pneumonia (POP) is a common complication after aneurysmal subarachnoid hemorrhage (aSAH) associated with increased mortality rates, prolonged hospitalization, and high medical costs. It is currently understood that identifying pneumonia early and implementing aggressive treatment can significantly improve patients' outcomes. The primary objective of this study was to explore risk factors and develop a logistic regression model that assesses the risks of POP.

**Methods:**

An internal cohort of 613 inpatients with aSAH who underwent surgery at the Neurosurgical Department of First Affiliated Hospital of Wenzhou Medical University was retrospectively analyzed to develop a nomogram for predicting POP. We assessed the discriminative power, accuracy, and clinical validity of the predictions by using the area under the receiver operating characteristic curve (AUC), the calibration curve, and decision curve analysis (DCA). The final model was validated using an external validation set of 97 samples from the Medical Information Mart for Intensive Care IV (MIMIC-IV) database.

**Results:**

Among patients in our internal cohort, 15.66% (*n* = 96/613) of patients had POP. The least absolute shrinkage and selection operator (LASSO) regression analysis identified the Glasgow Coma Scale (GCS), mechanical ventilation time (MVT), albumin, C-reactive protein (CRP), smoking, and delayed cerebral ischemia (DCI) as potential predictors of POP. We then used multivariable logistic regression analysis to evaluate the effects of these predictors and create a final model. Eighty percentage of patients in the internal cohort were randomly assigned to the training set for model development, while the remaining 20% of patients were allocated to the internal validation set. The AUC values for the training, internal, and external validation sets were 0.914, 0.856, and 0.851, and the corresponding Brier scores were 0.084, 0.098, and 0.143, respectively.

**Conclusion:**

We found that GCS, MVT, albumin, CRP, smoking, and DCI are independent predictors for the development of POP in patients with aSAH. Overall, our nomogram represents a reliable and convenient approach to predict POP in the patient population.

## Introduction

According to the Global Burden of Diseases, Injuries, and Risk Factors Study (GBD), the annual number of strokes and deaths due to stroke increased substantially from 1990 to 2019 (1). Following the methodology in the GBD, in 2019, there were 3.94 million (95% uncertainty interval 3.43–4.58) new stroke cases in China. The incidence rate of stroke increased by 86.0% (73.2–99.0) from 1990, reaching 276.7 (241.3–322.0) per 100,000 population in 2019 (2). In addition, since 2015, stroke has become the leading cause of death and disability in China, posing a significant threat to the health of its citizens as a major chronic non-communicable disease (3). Therefore, stroke-related research is very much needed. Aneurysmal subarachnoid hemorrhage (aSAH) is a serious condition frequently caused by intracranial aneurysm rupture and is associated with high rates of morbidity and mortality (4). The incidence of aSAH is estimated to be ~9.1 cases per 100,000 individuals, accounting for ~5% of all strokes (5, 6). Although the advent of endovascular coil embolization and microsurgery represents significant improvements in treating aSAH patients, postoperative complications remain challenging, affecting patient prognosis and increasing hospitalization costs. Postoperative pneumonia (POP) is a common complication affecting 13.2–29.15% of aSAH patients and is associated with prognosis (7–11). Therefore, this research sought to identify early risk factors for POP, which can help identify high-risk patients who may benefit from proactive monitoring and therapeutic interventions. Previous studies have shown that increased incidence of POP in aSAH patients was associated with factors such as high preoperative inflammatory markers, high levels of lactate dehydrogenase (LDH), the use of mechanical ventilation (MV), and high World Federation of Neurosurgical Societies (WFNS) grade (8, 12). While several risk factors for POP have been identified, predicting its occurrence in patients with aSAH remains challenging. To address this issue, one potential solution is to construct a mathematical model for predicting the probability of occurrence of POP. A nomogram, a graphical representation of regression models, can simplify risk assessment, map the probability of events to individual patients, and enhance clinical decision-making for medical staff and patients (13). This retrospective study aimed to identify the preoperative factors associated with POP and develop a nomogram based on perioperative information to predict the likelihood of POP, providing clinicians with a useful tool for preventing and treating this patient population.

## Materials and methods

### Data source

This retrospective study consisted of two cohorts: an internal cohort (*n* = 613) and an external cohort (*n* = 97). A total of 613 adult patients from the internal cohort were newly diagnosed with aSAH between 1 January 2019 and 5 February 2022 at the First Affiliated Hospital of Wenzhou Medical University. The study samples and treatment information were obtained from the database of the neurosurgical departments. The blood laboratory examination was collected within 24 h after patient's admission and before the surgery. The external cohort (*n* = 97) was extracted from the Medical Information Mart for Intensive Care IV (MIMIC-IV) (version 2.2) database, derived from a large, freely accessible critical care database comprising 299,712 patients who were admitted to the ICU or the emergency department of Beth Israel Deaconess Medical Center between 2008 and 2019 (https://mimic.physionet.org/) (14, 15).

### Patient enrollment

Patients enrolled in the internal cohort met the following requirements: (1) aged 18 years or older, experiencing their first-ever subarachnoid hemorrhage, and admitted to the hospital within 24 h of symptoms onset; (2) received confirmation of aSAH through computed tomography (CT), computed tomographic angiography (CTA), or digital subtraction angiography (DSA); and (3) underwent embolization of intracranial aneurysms. Some of them also received intracranial stenting and/or craniotomy. Among the patients that met the above conditions (*n* = 763), 150 were excluded for the following reasons: (1) the presence of other potential causes of SAH [arteriovenous malformation (*n* = 90), craniocerebral trauma (*n* = 3), and hypertensive intracerebral hemorrhage (*n* = 2)]; (2) having a history of malignant tumors, severe heart, hepatic, or renal failure (*n* = 10); (3) previous use of antibiotics, systemic glucocorticoids, immunosuppressive agents, or immunotherapy within 1 month before admission (*n* = 3); (4) diagnosed with pneumonia according to the modified Centers for Disease Control and Prevention (CDC) criteria before admission (*n* = 30) (9, 16); and (5) died within 24 h after surgery or incomplete records (*n* = 12). Finally, 613 patients were included in the internal cohort, with 80% randomly assigned to the training set for model development. The remaining 20% constituted the validation set that was used to evaluate the nomogram model.

**Figure 1:**
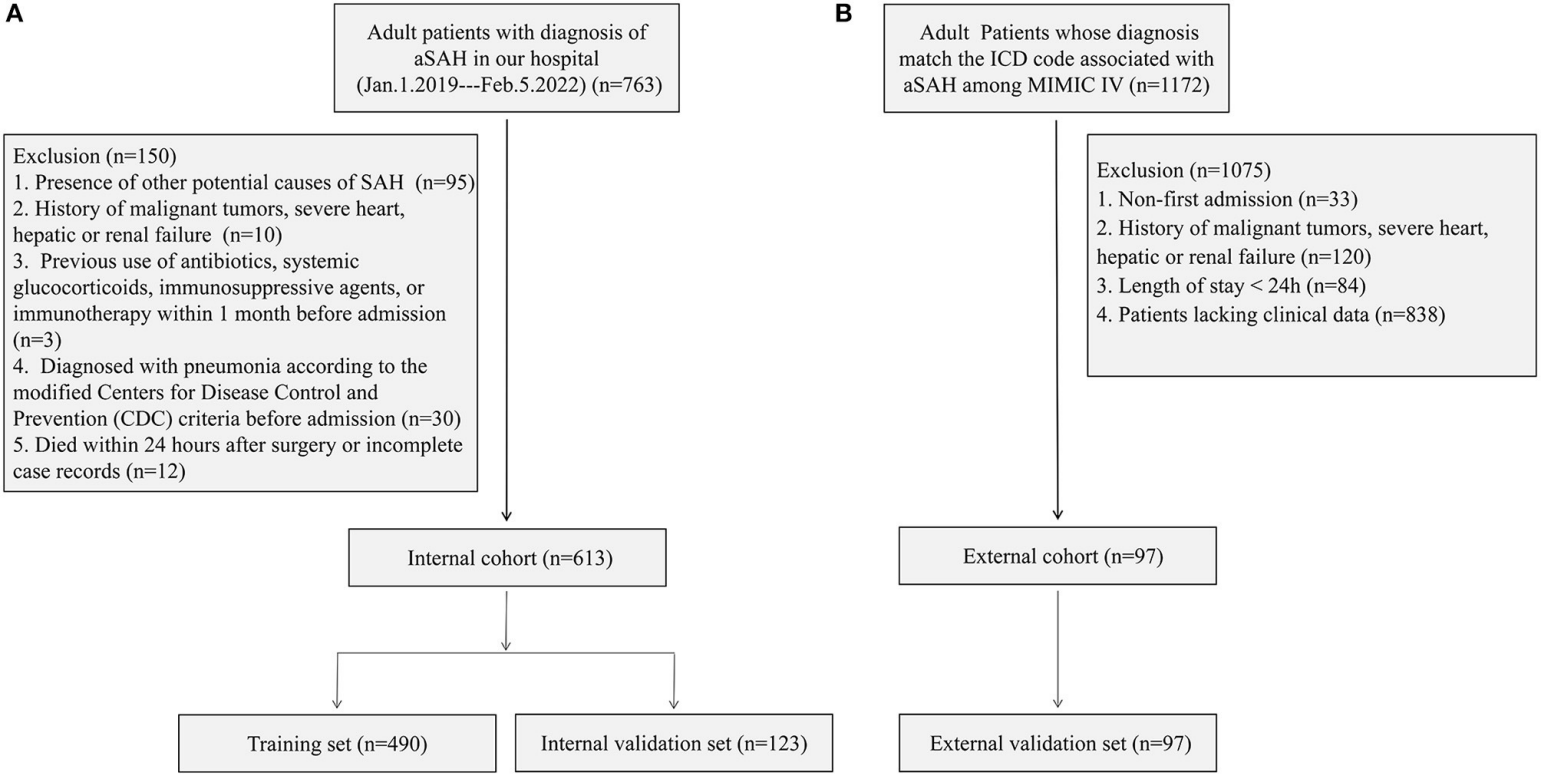
Flowchart of the internal cohort **(A)** and external cohort **(B)**.

In the external cohort, the inclusion criteria were as follows: (1) patients aged 18 years or older and (2) those whose diagnosis matches the International Classification of Diseases (ICD) code associated with aSAH among MIMIC-IV (ICD-9 code 430, and ICD-10 codes I60 to I609). A total of 1,172 patients met the above conditions. The exclusion criteria were as follows: (1) non-first admission (*n* = 33); (2) a history of malignant tumors, severe heart, hepatic, or renal failure (*n* = 120); (3) length of stay of < 24 h (*n* = 84); and (4) patients lacking clinical data (*n* = 838). Finally, 97 patients were assigned to the external validation set to validate the nomogram. Figure 1 shows the detailed flowchart.

According to the modified CDC criteria (16), POP was defined as the set of lower respiratory tract infections that occur within 30 days after an operative procedure, which pertains to the following: (1) A probable POP could not be diagnosed based on the admission or the follow-up chest x-ray, and it could neither be explained by another diagnosis and (2) proven POP had a confirmed change in diagnosis on at least one image of the chest x-ray. Patients with probable/proven pneumonia were considered cases for this study by the modified CDC criteria (17, 18), whereas all patients with pneumonia before admission were excluded.

### Variable selection and nomogram construction

In this study, various clinical data were collected on the internal cohort, including age, gender, smoking, drinking, aneurysm location [anterior communicating artery (ACoA), internal carotid artery (ICA), middle cerebral artery (MCA), posterior communicating artery (PCoA), vertebrobasilar artery (VBA)], the Hunt and Hess Scale grades, the modified Fisher Scale (mFS), the Glasgow Coma Scale (GCS), hypertension, chronic obstructive pulmonary disease (COPD), coronary heart disease (CHD), diabetes, delayed cerebral ischemia (DCI), deep vein thrombosis (DVT), POP, intracranial stenting, with craniotomy, mechanical ventilation time (MVT), neutrophil count, monocyte count, lymphocyte count, hemoglobin, blood platelet count, mean corpuscular volume (MCV), albumin, glucose, triglyceride, total cholesterol, uric acid, D-dimer, and C-reactive protein (CRP). The optimal predictive variables were selected using the least absolute shrinkage and selection operator (LASSO) regression. Multivariable logistic regression analysis was then generated using selected predictors from LASSO analysis to develop a nomogram for predicting the risks of POP in aSAH patients.

The nomogram's predictive performance was validated using an external cohort of patients from the MIMIC-IV database. The data of the external cohort included POP and six final predictors: GCS, MVT, albumin, CRP, smoking, and DIC.

### Statistical analysis

In this study, continuous variables were described in terms of mean ± SD or median with the interquartile range (IQR), while categorical variables were expressed as frequency and percentage. A LASSO regression model was used to select the optimal predictive variables to address collinearity among the candidate variables (19). Predictors selected from the LASSO analysis were used to generate a multivariate logistic regression analysis, where the features were represented by odds ratio (OR) and 95% confidence intervals (CIs). The receiver operating characteristic curve (ROC) was plotted, and the area under the ROC curve (AUC) was determined to evaluate the discrimination of the nomogram. Decision curve analysis (DCA) was used to determine the nomogram's clinical validity and net benefit (20). The calibration of the nomogram model was evaluated using the Brier score and the calibration curve, with 1,000 bootstrap resamples employed. A two-sided *P*-value of < 0.05 was considered to be statistically significant.

## Results

### Population characteristics

Table 1 shows the clinical characteristics of the study population derived from the internal cohort. A total of 613 patients were included in this study and divided into a training set (*n* = 490) and an internal validation set (*n* = 123). The overall incidence of POP in the internal cohort was ~15.66%.

**Table 1:** Characteristics of the internal cohort.

**Characteristics**	**Internal set (*n =* 613)**	**Training set (*n =* 490)**	**Internal validation set (*n =* 123)**	***p***
**Demographics**				
Age (years), median [IQR]	55.00 [48.00, 66.00]	55.00 [48.00, 66.00]	53.00 [48.00, 64.00]	0.233
Gender, *n* (%)				0.755
Men	207 (33.77)	164 (33.47)	43 (34.96)	
Women	406 (66.23)	326 (66.53)	80 (65.04)	
Smoking, *n* (%)	73 (11.91)	61 (12.45)	12 (9.76)	0.410
Drinking, *n* (%)	86 (14.03)	72 (14.69)	14 (11.38)	0.344
**Aneurysm location**				
ACoA, *n* (%)	195 (31.81)	165 (33.67)	30 (24.39)	0.048
ICA, *n* (%)	183 (29.85)	145 (29.59)	38 (30.89)	0.778
MCA, *n* (%)	91 (14.85)	72 (14.69)	19 (15.45)	0.834
PCoA, *n* (%)	129 (21.04)	100 (20.41)	29 (23.58)	0.441
VBA, *n* (%)	44 (7.18)	36 (7.35)	8 (6.50)	0.746
**Scoring systems**				
Hunt Hess grade, *n* (%)				0.258
Grade 1, 2, 3	529 (86.30)	419 (85.51)	110 (89.43)	
Grade 4, 5	84 (13.70)	71 (14.49)	13 (10.57)	
mFS, *n* (%)				0.141
Grade 1, 2	425 (69.33)	333 (67.96)	92 (74.80)	
Grade 3, 4	188 (30.67)	157 (32.04)	31 (25.20)	
GCS, median [IQR]	15.00 [15.00, 15.00]	15.00 [14.00, 15.00]	15.00 [15.000, 15.00]	0.264
**Comorbidities**				
Hypertension, *n* (%)	309 (50.41)	241 (49.18)	68 (55.29)	0.226
COPD, *n* (%)	5 (0.82)	4 (0.82)	1 (0.81)	0.997
CHD, *n* (%)	18 (2.94)	14 (2.86)	4 (3.25)	0.817
Diabetes, *n* (%)	44 (7.18)	34 (6.94)	10 (8.13)	0.647
DCI, *n* (%)	44 (7.18)	35 (7.14)	9 (7.32)	0.947
DVT, *n* (%)	57 (9.30)	49 (10.00)	8 (6.50)	0.233
POP, *n* (%)	96 (15.66)	78 (15.92)	18 (14.63)	0.726
**Treatments**				
Intracranial stenting, *n* (%)	247 (40.29)	201 (41.02)	46 (37.40)	0.464
With craniotomy, *n* (%)	115 (18.76)	96 (19.59)	19 (15.45)	0.292
MVT, *n* (%)				0.937
Short-term ventilation (< 96 h)	69 (11.26)	56 (11.43)	13 (10.57)	
Long-term ventilation (≥96 h)	73 (11.91)	59 (12.04)	14 (11.38)	
**Laboratory tests**				
Neutrophil count (× 10^9^ /L), median [IQR]	9.61 [6.96, 12.63]	9.47 [6.92, 12.47]	10.08 [7.72, 13.24]	0.106
Monocyte count (× 10^9^ /L), median [IQR]	0.47 [0.31, 0.70]	0.47 [0.32, 0.70]	0.44 [0.28, 0.71]	0.672
Lymphocyte count (× 10^9^ /L), median [IQR]	1.14 [0.85, 1.54]	1.12 [0.82, 1.56]	1.16 [0.94, 1.54]	0.399
Hemoglobin (g/L), median [IQR]	132.0 [120.00, 142.00]	131.0 [120.00, 142.00]	133.0 [122.00, 144.00]	0.449
Platelet count (× 10^9^ /L), median [IQR]	212.0 [175.00, 256.00]	209.0 [172.00, 254.00]	229.0 [191.00, 264.00]	0.005
MCV (fl), median [IQR]	89.80 [86.80, 92.60]	89.80 [86.80, 92.60]	89.50 [86.30, 92.80]	0.736
Albumin (g/L), median [IQR]	39.00 [36.00, 41.50]	38.90 [36.00, 41.50]	39.00 [36.90, 41.70]	0.278
Glucose (mmol /L), median [IQR]	6.40 [5.40, 7.60]	6.400 [5.40, 7.60]	6.50 [5.30, 7.80]	0.645
Triglyceride (μmol /L), median [IQR]	1.08 [0.82, 1.69]	1.10 [0.84, 1.70]	0.99 [0.73, 1.50]	0.119
Total cholesterol (μmol /L), median [IQR]	4.88 [4.30, 5.66]	4.87 [4.30, 5.65]	4.95 [4.41, 5.81]	0.453
Uric acid (μmol /L), median [IQR]	241.0 [174.00, 299.00]	241.0 [173.00, 296.00]	236.0 [185.00, 315.00]	0.758
D-dimer (mg/L), median [IQR]	1.15 [0.56, 2.56]	1.15 [0.56, 2.55]	1.20 [0.53, 2.75]	0.879
CRP (mg/L), median [IQR]	6.30 [2.90, 15.80]	6.36 [2.90, 15.80]	6.00 [1.60, 15.70]	0.206

IQR, interquartile range; ACoA, anterior communicating artery; ICA, internal carotid artery; MCA, middle cerebral artery; PCoA, posterior communicating artery; VBA, vertebrobasilar artery; mFS, modified Fisher Scale; GCS, Glasgow Coma Scale; COPD, chronic obstructive pulmonary disease; CHD, coronary heart disease; DCI, delayed cerebral ischemia; DVT, deep vein thrombosis; POP, postoperative pneumonia; MVT, mechanical ventilation time**;** MCV, mean corpuscular volume; CRP, C-reactive protein.

Table 2 shows the characteristics of six final predictors and POP in the internal and external cohorts. The overall incidence of POP in the external cohort was ~20.62%, which is similar to the internal cohort.

**Table 2:** Characteristics of POP and six final predictors in the internal and external cohorts.

**Characteristics**	**All patients (*n =* 710)**	**Internal cohort (*n =* 613)**	**External cohort (*n =* 97)**	***P*-value**
POP, *n* (%)	116 (16.34)	96 (15.66)	20 (20.62)	0.22
GCS, median [IQR]	15.00 [13.00, 15.00]	15.00 [15.00, 15.00]	14.00 [13.00, 14.00]	< 0.001
MVT, *n* (%)				0.135
Short-term ventilation (< 96 h)	86 (12.11)	69 (11.26)	17 (17.53)	
Long-term ventilation (≥96 h)	87 (12.25)	73 (11.91)	14 (14.43)	
Albumin (g/L), median [IQR]	38.80 [36.00, 41.50]	39.00 [36.00, 41.50]	37.40 [34.00, 41.00]	0.049
CRP (mg/L), median [IQR]	6.50 [2.90, 19.20]	6.30 [2.90, 15.80]	14.80 [3.80, 57.80]	< 0.001
Smoking, *n* (%)	97 (13.66)	73 (11.91)	24 (24.74)	< 0.001
DCI, *n* (%)	61 (8.59)	44 (7.18)	17 (17.53)	< 0.001

POP, postoperative pneumonia; GCS, Glasgow Coma Scale; MVT, mechanical ventilation time**;** CRP, C-reactive protein; DCI, delayed cerebral ischemia.

Table 3 shows the length of stay of aSAH patients with POP and non-POP in the internal cohort. The median length of hospital stay in patients with POP was 20 days, which was significantly higher than the 13 days in patients with non-POP (*P* < 0.001).

**Table 3:** Length of stay of patients with POP and non-POP with aSAH in the internal cohort.

**Variable**	**All patients (*n =* 613)**	**POP negative (*n =* 517)**	**POP positive (*n =* 96)**	***P*-value**
Length of stay, median [IQR]	14.00 [10.00, 19.00]	13.00 [10.00, 18.00]	20.00 [15.00, 28.00]	< 0.001

POP, postoperative pneumonia.

### Selected predictors

**Figure 2:**
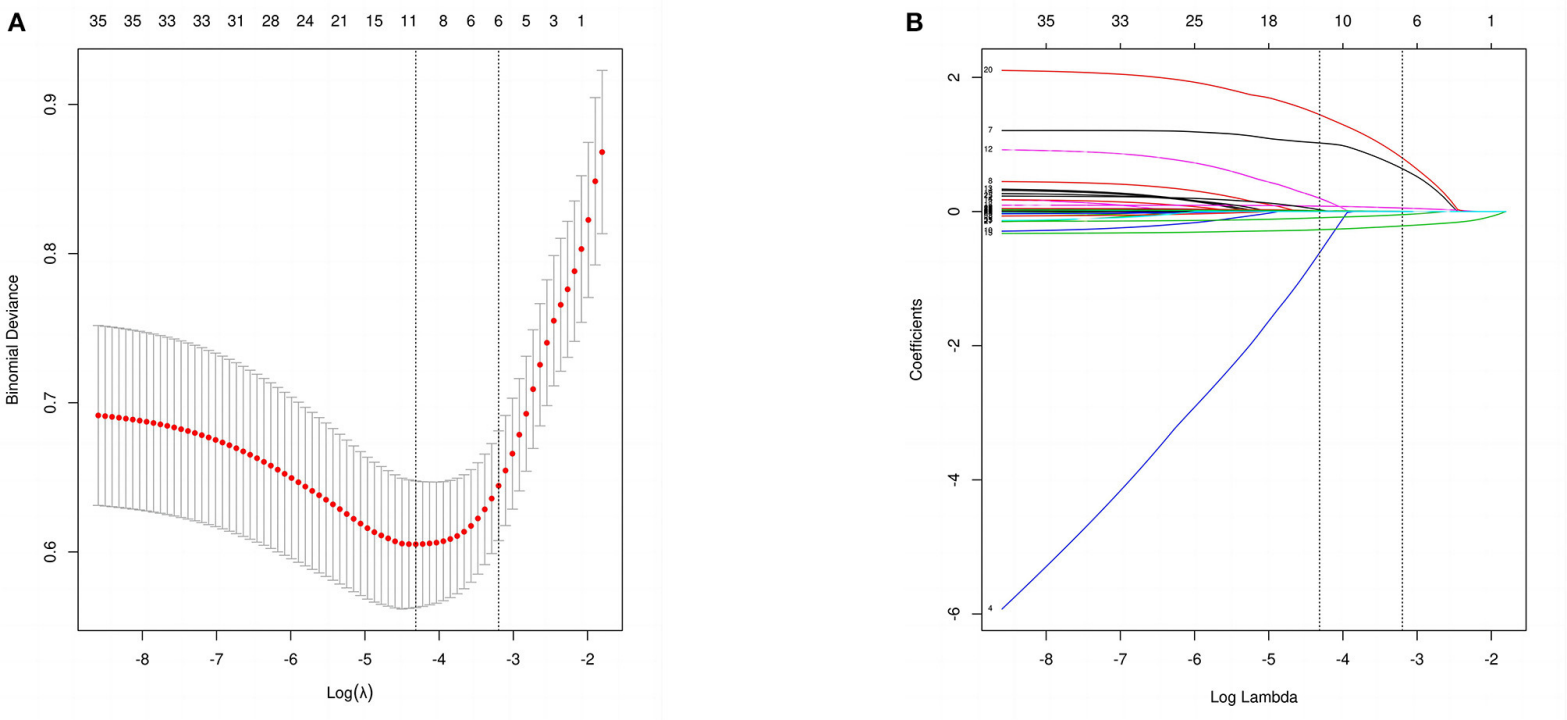
Perioperative variable selection using a LASSO logistic regression model. **(A)** The minimum criteria (lambda.min) and 1 SE of the minimum criteria (lambda.1se) were used to depict the optimal values with dotted vertical lines. **(B)** LASSO coefficient profile of 34 variables. The coefficient profile is plotted according to the logarithmic sequence. To determine the optimal predictors of the model, five-fold cross-validation with minimum criteria was used, resulting in six features with nonzero coefficients.

Out of 34 independent variables, 6 potential predictors were selected by using LASSO regression analysis in the training set (Figure 2), including GCS, MVT, albumin, CRP, smoking, and DIC. A final model was created using multivariable logistic regression analysis based on six predictors screened from LASSO regression analysis (Table 4). In multivariable logistic regression, the influence of these six independent variables on the dependent variable POP was statistically significant (*P* < 0.05).

**Table 4:** Prediction factors for the risk of postoperative pulmonary infection with aSAH.

**Predictor**	**Estimate**	**SE**	**Z**	***P*-value**	**Odds ratio**	**Lower**	**Upper**
(Intercept)	5.418	1.314	4.124	< 0.001	225.488	18.11	3201.889
GCS	−0.352	0.052	−6.725	< 0.001	0.703	0.632	0.777
MVT	0.088	0.034	2.579	0.01	1.092	1.021	1.167
Albumin	−0.098	0.028	−3.502	< 0.001	0.906	0.857	0.957
CRP	0.023	0.007	3.289	0.001	1.024	1.01	1.038
Smoking	2.002	0.393	5.094	< 0.001	7.403	3.428	16.119
DCI	1.535	0.486	3.159	0.002	4.641	1.774	12.025

GCS, Glasgow Coma Scale; MVT, mechanical ventilation time**;** CRP, C-reactive protein; DCI, delayed cerebral ischemia.

### Construction and validation of the nomogram

**Figure 3:**
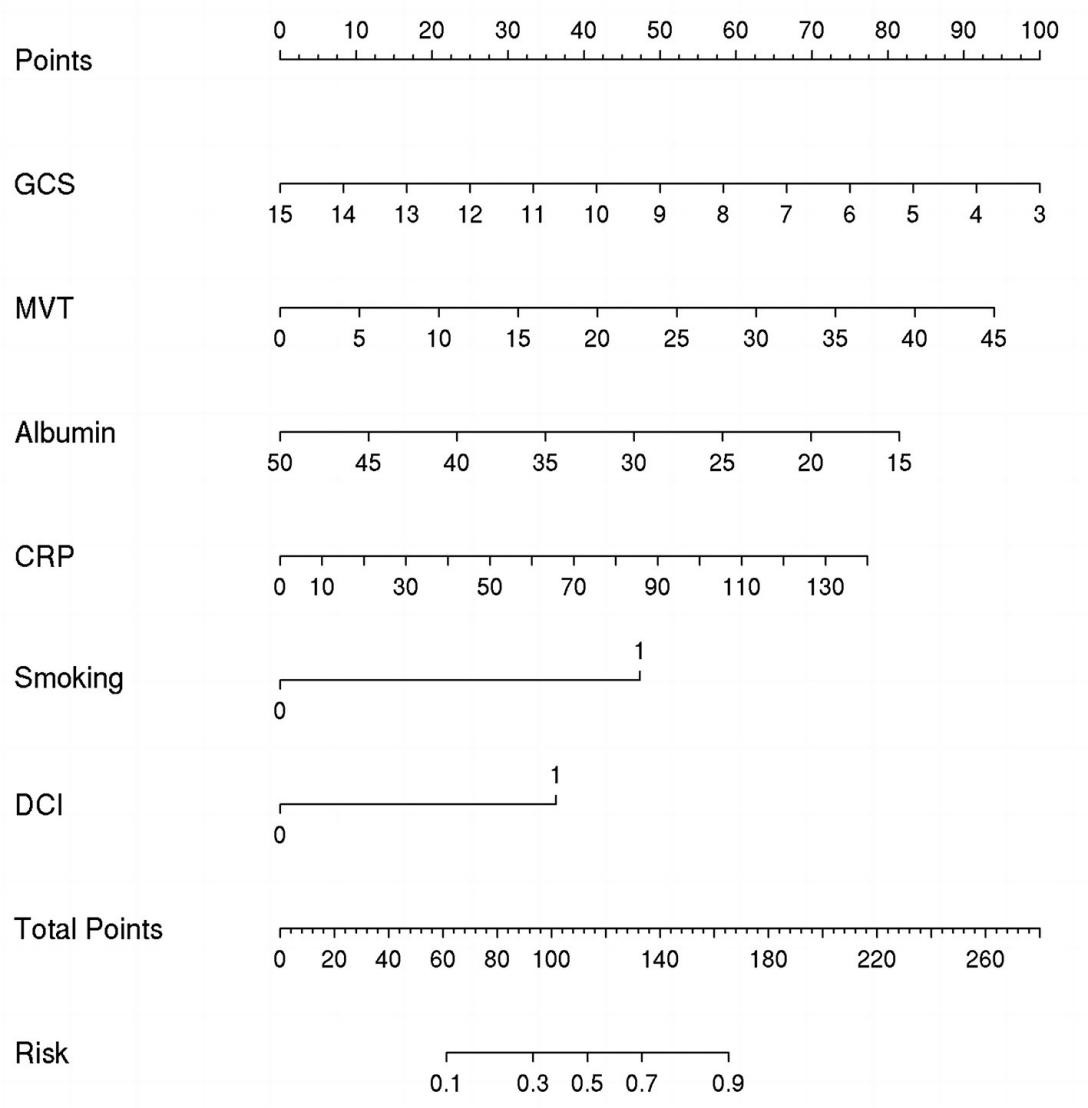
Nomogram for predicting the risk of postoperative pneumonia (POP). Points were assigned for Glasgow Coma Scale (GCS), mechanical ventilation time (MVT), albumin, C-reactive protein (CRP), smoking, and delayed cerebral ischemia (DCI). The total score obtained by adding up the scores of all individual variables is used to find the appropriate position on the “Risk of POP” axis to determine the patient's individual risk of POP.

The nomogram presented in Figure 3 could predict the likelihood of POP in patients with aSAH. The model considers several factors, with GCS having the greatest effect on the development of POP, followed by MVT, albumin, CRP, smoking, and DCI. The total score of these independent prognostic factors was positively correlated with the patient's risk of POP.

**Figure 4:**
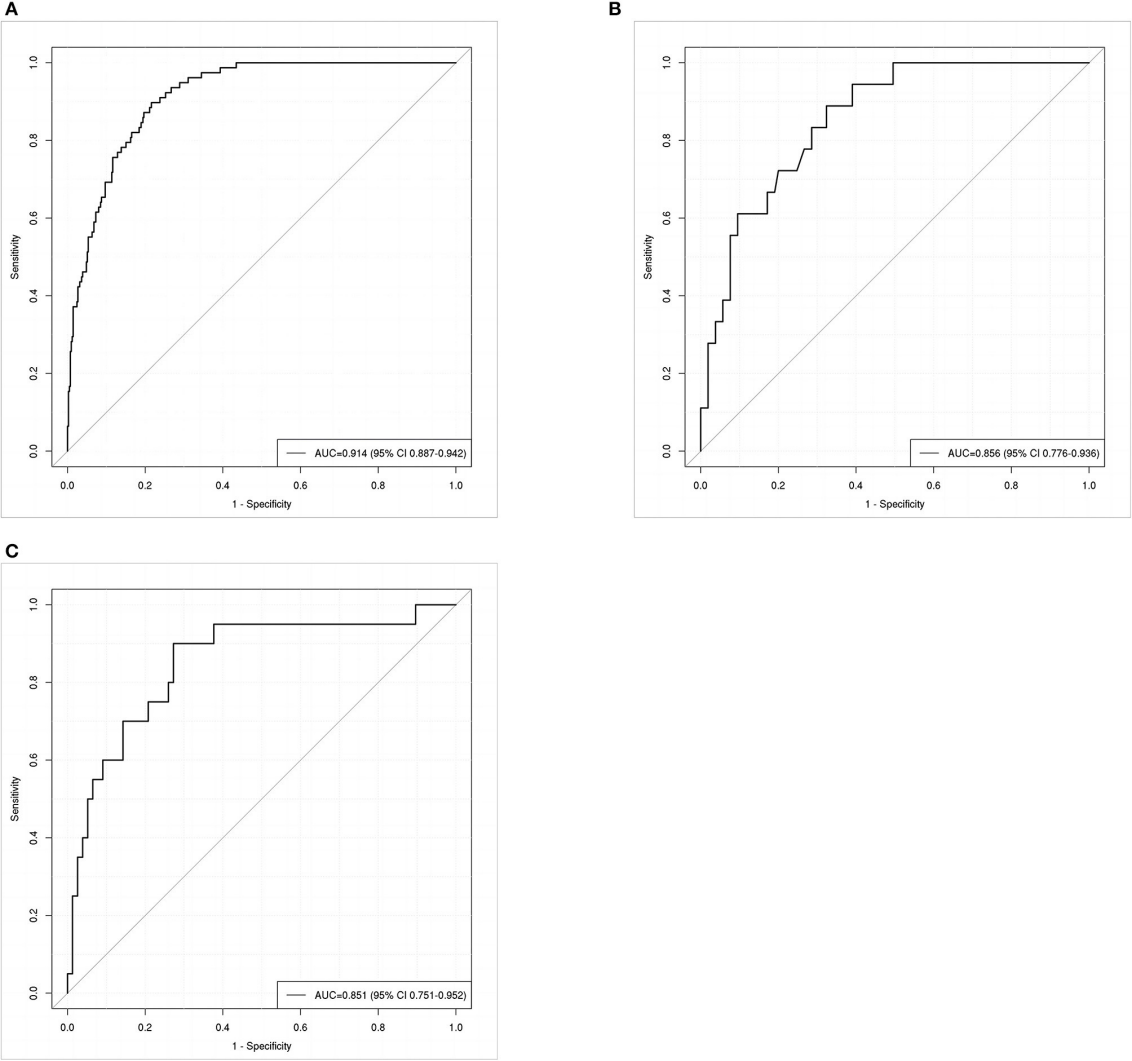
Receiver operating characteristic (ROC) curves for predicting postoperative pneumonia in aneurysmal subarachnoid hemorrhage patients in the training set **(A)**, internal validation set **(B)**, and external validation set **(C)**. The area under the receiver operating characteristic curve (AUC) of the training set **(A)**, internal validation set **(B)**, and external validation set **(C)** was 0.914, 0.856, and 0.851, respectively.

ROC curve analysis was conducted to evaluate the nomogram model's discrimination performance. The AUC value was 0.914 (95% CI: 0.887–0.942, Figure 4A) in the training set and 0.856 (95% CI: 0.776–0.936, Figure 4B) in the internal validation set. We further assessed the model's performance using an external validation set which yielded an AUC of 0.851 (95% CI: 0.751–0.952, Figure 4C), indicating the good discrimination performance of the nomogram.

**Figure 5:**
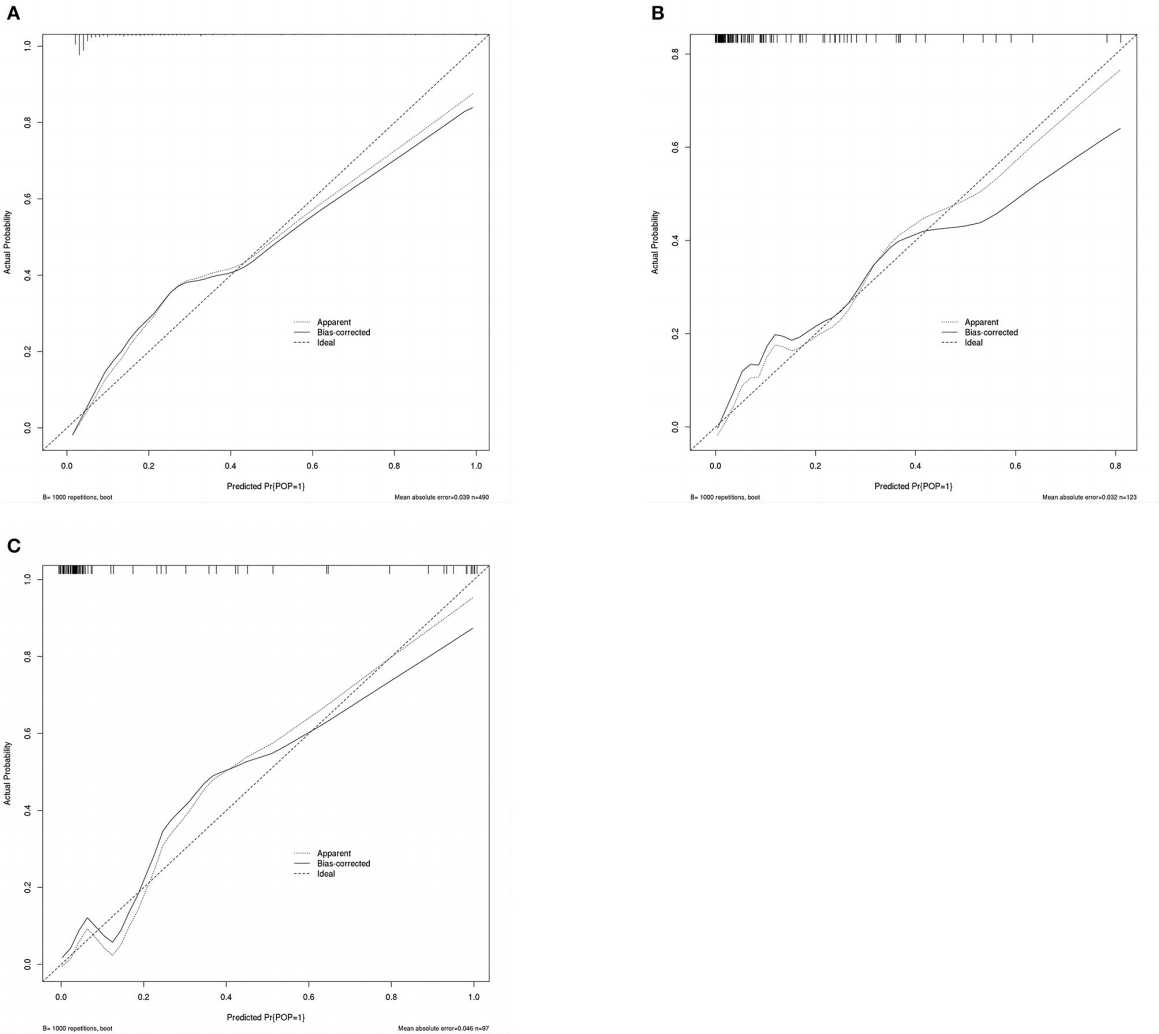
Calibration plot of the nomogram in the training set **(A)**, internal validation set **(B)**, and external validation set **(C)**. Predictions generated from the model are plotted against actual patient outcomes. The 45-degree line represents the perfect model calibration. The dotted line (apparent) indicates calibration when the model is applied to each set, and the solid line (bias-corrected) indicates calibration when the model is applied to the bootstrap set.

**Figure 6:**
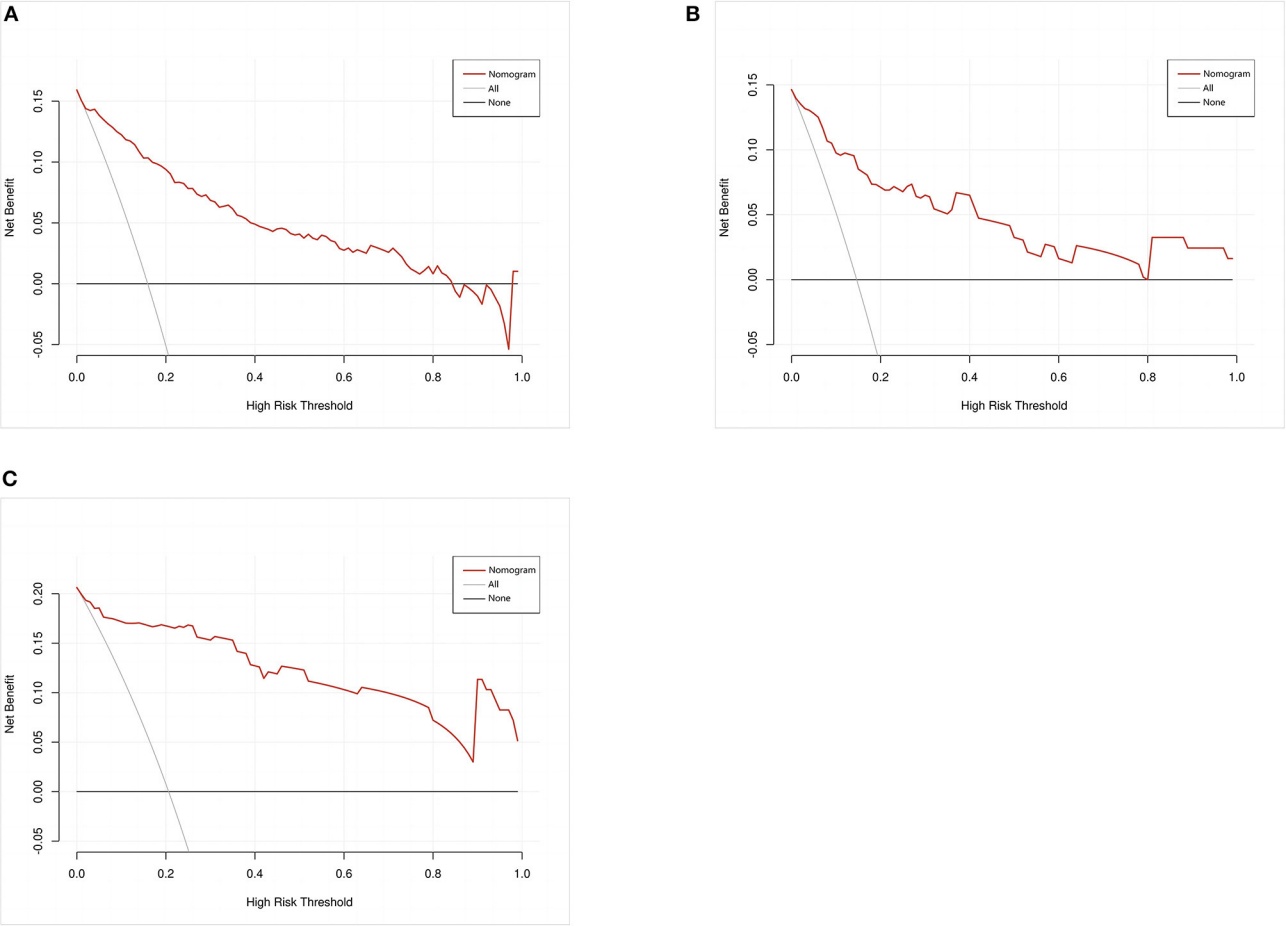
Decision curve analysis of the nomogram in the training set **(A)**, internal validation set **(B)**, and external validation set **(C)**. The red line displays the net benefit of our model. The gray line assumes that all patients develop postoperative pneumonia (POP). The black line assumes that no patients develop POP.

A calibration plot was constructed by using 1,000 bootstrap resamples to assess the nomogram's calibration performance. The results showed an optimal agreement between the predicted and observed outcomes in the training set (Figure 5A), the internal validation set (Figure 5B), and the external validation set (Figure 5C). The mean absolute error values for the three calibration plots were 0.039, 0.032, and 0.046, respectively. To quantify the performance of calibration, we utilized the Brier score. The results showed that the Brier scores were 0.084 in the training set, 0.098 in the internal validation set, and 0.143 in the external validation set. Additionally, we conducted a DCA to evaluate the clinical benefit of the nomogram. As illustrated in Figures 6A–C, decision curve analyses of the training set, the internal validation set, and the external validation set demonstrated a net clinical benefit.

## Discussion

In this observational study, GCS, MVT, albumin, CRP, smoking, and DCI were identified as independent predictors of POP in patients with aSAH. Using these six variables, we constructed a nomogram to assess the risk of POP in this patient population. During internal validation, we successfully demonstrated that the model exhibited both discriminative and well-calibrated performance. Although there may be differences between the internal database and the external database due to race, time of admission and discharge of patients, and confounding factors, external validation confirmed its satisfactory accuracy and generalizability.

This study analyzed the incidence of POP in patients with aSAH from the internal cohort and found it to be ~5.66% lower than that reported in most previous cases (7–11, 21), attributed to the exclusion of patients with pneumonia before operation and advancements in surgical techniques and perioperative care.

Our study found that GCS had the greatest impact on the development of POP according to the nomogram. Patients who developed POP had significantly lower GCS scores compared to those who did not develop POP (*P* < 0.01), which could be attributed to lower GCS scores being associated with decreased consciousness, limb movement disorders, and prolonged bedridden periods, which can result in stroke-associated pneumonia (SAP) (22). Additionally, GCS is a reliable indicator of unfavorable neurological outcomes (23).

Herein, we found that MVT was an independent risk factor for POP (*P* = 0.001, OR = 1.108, 95% CI: 1.044–1.079). Previous research has demonstrated that prompt extubation could significantly decrease the likelihood of POP. A study conducted by Vera Urquiza et al. (21) discovered that extubation after 6 h was an independent risk factor for pneumonia following cardiac surgery (*P* = 0.005, OR = 15.81, 95% CI: 2.2–110.7). In a study by Savardekar et al. (11), it was found that tracheal intubation lasting over 48 h increased the risk of POP in patients undergoing microsurgical clipping of ruptured intracranial aneurysms (*P* = 0.041, OR = 6.638, 95% CI: 1.08–40.8) likely due to the association between long-term intubation and ventilator-associated pneumonia (24). Therefore, daily sedation interruption and assessment of readiness to extubate can reduce the risk of ventilator-associated pneumonia and prevent pneumonia in these patients.

Consistent with the literature, albumin was a major predictor for POP in aSAH patients (9). Furthermore, other studies have demonstrated that albumin therapy can improve organ function and reduce complications in aSAH patients, potentially leading to better outcomes (25–27). This finding may be attributed to the fact that low levels of albumin in the blood can cause a decrease in plasma osmotic pressure, leading to pulmonary interstitial edema. Additionally, hypoalbuminemia can weaken the immune system of patients, increasing their susceptibility to pulmonary infections (28).

CRP is a well-known biomarker of systemic inflammation (29). An increasing body of evidence suggests that CRP can predict various postoperative infections (30–32). Our study also indicated that monitoring serum CRP levels can be useful in predicting POP in patients with aSAH.

Moreover, we found that smoking was identified as an independent risk factor for POP in aSAH patients. Similarly, it is reportedly a risk factor for pneumonia after lung cancer surgery (33), attributed to the inhibitory effect of smoking on the innate immune system. Previous reviews have suggested that quitting smoking after early-stage lung cancer can improve the prognosis (34). Therefore, quitting smoking may also provide benefits for aSAH patients.

Our study found that DCI was independently associated with the development of POP in patients with aSAH. However, further investigation into the temporal course of DCI and its relationship with the development of POP is necessary to establish causation. It is highly conceivable that causation is not a linear process but rather a result of intricate interactions between dynamic systems (35). On the one hand, infection is a known cause of systemic inflammation, which can lead to an increase in platelets (thrombocytosis) (36), white blood cells (leukocytosis), and the release of inflammatory cytokines—specifically interleukin-6 (37). All these factors have been linked to the development of DCI (38–41). On the other hand, DCI has been associated with lower levels of consciousness, low GCS score, and prolonged bedridden periods, which leads to stroke-associated pneumonia (SAP) (22).

Several limitations of this study should be acknowledged. First, the study's retrospective nature introduces the possibility of confounding factors influencing the results. Although the findings have been validated in another database, conducting a large prospective controlled trial and validating several different external datasets are crucial to validate the reported results. Second, several risk factors reported by previous studies were not involved in this study, such as WFNS grade (12) and high LDH (8). Third, patients were not followed up after discharge, indicating the need for longer-term studies to validate our findings. Fourth, in external cohort inclusion, it is difficult to find out whether patients have undergone surgery due to the lack of data. Therefore, the operation was not taken as the inclusion criteria for enrolling an external cohort. Furthermore, due to the limited information in the external database, we cannot exclude the external cohort using exactly the same criteria as the internal cohort. Not applying the same inclusion and exclusion criteria was also a factor affecting the results.

## Conclusion

Our findings corroborate that GCS, MVT, albumin, CRP, smoking, and DCI are important predictors of postoperative infection and enable physicians to make informed decisions and implement appropriate clinical interventions to reduce the risk of POP. Overall, our nomogram allows for accurate and straightforward prediction of pulmonary infection occurrence in this patient population.

## Data availability statement

The raw data supporting the conclusions of this article will be made available by the authors, without undue reservation.

## Ethics statement

The studies involving human participants were reviewed and approved by the Ethics Committee in Clinical Research (ECCR) of the First Affiliated Hospital of Wenzhou Medical University. The acceptance number of our review is KY2023-R079. Informed consent was not required in this study because the data collected did not contain personal identifiers.

## Author contributions

CC made substantial contributions to the conception and design of this study and agreed to be accountable for all aspects of the study in ensuring that questions related to the accuracy or integrity of any part of the study were appropriately investigated and resolved. XJ participated in drafting and revising the manuscript and in acquiring, collecting, analyzing, and interpreting the data. SW, CZ, and SY participated in collecting and interpreting the data. LL and SX participated in editing the final draft and confirmed the authenticity of the data. All authors have agreed to submit the article to the current journal, approved the final version for publication, and have taken accountability for all aspects of the article.
